# In Vitro Enhanced Skin Permeation and Retention of Imiquimod Loaded in β-Cyclodextrin Nanosponge Hydrogel

**DOI:** 10.3390/pharmaceutics11030138

**Published:** 2019-03-20

**Authors:** Monica Argenziano, Adam Haimhoffer, Chiara Bastiancich, László Jicsinszky, Fabrizio Caldera, Francesco Trotta, Sara Scutera, Daniela Alotto, Mara Fumagalli, Tiziana Musso, Carlotta Castagnoli, Roberta Cavalli

**Affiliations:** 1 Department of Drug Science and Technology, University of Turin, via P. Giuria 9, 10125 Turin, Italy; monica.argenziano@unito.it (M.A.); ljicsinszky@gmail.com (L.J.); 2 Department of Pharmaceutical Technology, Faculty of Pharmacy, University of Debrecen, H-4033 Debrecen, Hungary; adamhaimhoffer@gmail.com; 3 Aix-Marseille Univ, CNRS, INP, Inst Neurophysiopathol, 13344 Marseille, France; chiara.bastiancich@gmail.com; 4 Department of Chemistry, University of Turin, via Giuria 7, 10125 Turin, Italy; fabrizio.caldera@unito.it (F.C.); francesco.trotta@unito.it (F.T.); 5 Dipartimento di Scienze della Sanità Pubblica e Pediatriche, University of Turin, 10126 Turin, Italy; sara.scutera@unito.it (S.S.); tiziana.musso@unito.it (T.M.); 6 Dipartimento di Chirurgia Generale e Specialistiche, Banca della Cute, AOU Città della Salute e della Scienza di Torino, 10126 Turin, Italy; daniela.alotto@gmail.com (D.A.); mara.fumagalli@gmail.com (M.F.); ccastagnoli@cittadellasalute.to.it (C.C.)

**Keywords:** cyclodextrins, inclusion complex, nanosponges, controlled release, imiquimod

## Abstract

Imiquimod (IMQ) is an immune response modifier clinically used for the treatment of various topical diseases. However, its poor aqueous solubility and skin penetration capability make the topical delivery of IMQ a challenging task. This work aims at developing a nanomedicine-based topical formulation, carrying IMQ to control the scarring process for the treatment of aberrant wounds. For this purpose, IMQ was loaded in β-cyclodextrin-based nanosponges and dispersed in a hydrogel suitable for dermal application. The formulation was characterized in vitro and compared with IMQ inclusion complexes, with (2-hydroxy)propyl β-cyclodextrin(HPβCD) and carboxymethyl β-cyclodextrin (CMβCD) showing enhanced penetration properties. The hydrogel containing IMQ-loaded nanosponges could act as a drug reservoir and guarantee the sustained release of IMQ through the skin. A greater inhibitory effect on fibroblast proliferation was observed for IMQ loaded in nanosponges compared to the other formulations.

## 1. Introduction

**Scheme 1:**
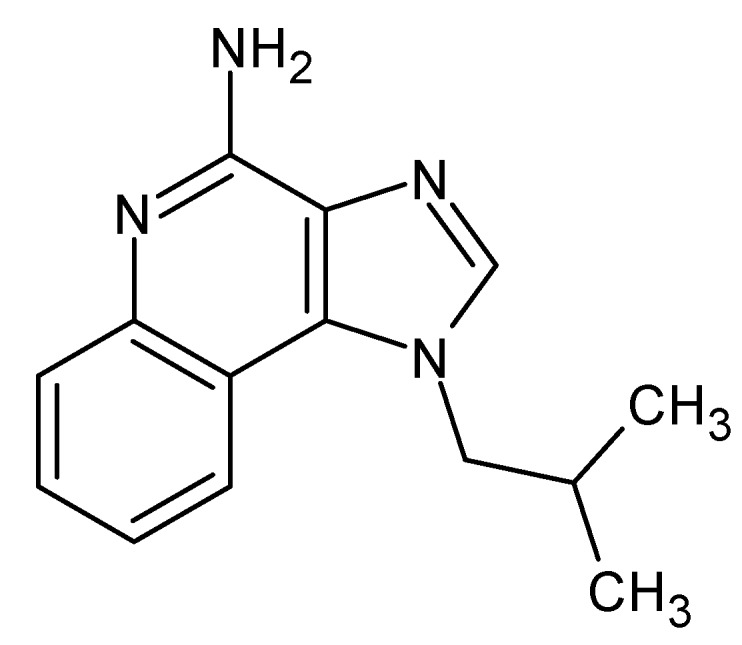
Imiquimod chemical structure.

Imiquimod (IMQ,1-[2-methylpropyl]-1*H*-imidazo [4,5-c]quinolin-4-amine; Scheme 1) is an immune response modifier able to stimulate the production of Interferon-α (IFN-α) and other proinflammatory cytokines, inducing a cell-mediated immune response and increased collagen breakdown. In addition, IMQ can modify the expression of genes associated with apoptosis [1,2] and is clinically used for the treatment of neoplastic skin diseases [3]. In 2004, the Food and Drug Administration (FDA) approved the 5% IMQ cream, Aldara^®^, for the treatment of actinic keratosis and superficial basal cell carcinoma in adults. Since then, the unlicensed use of IMQ has been extended to the treatment of different dermatological pathologies including hypertrophic scars (HS). HS are caused by uncontrolled and aberrant wound healing with excessive fibrosis and deposition of extracellular matrix [4].

In particular, the appearance of HS is estimated in about 70% of post-burn patients [5]. The presence of scars can contribute to neuropathic pain, stiffness and contractures, resulting in a reduced quality of life [6]. Therefore, scar management is a key factor for impairing patient rehabilitation and reintegration into society. 

Several approaches, either surgical or nonsurgical, have been studied to control skin scarring. Currently, pressure garments, silicone or non-silicone gel absorbent and pharmacological therapy are used without showing a significant improvement on the patient’s quality of life. Recently, advanced strategies such as cell therapy, gene delivery and nanomedicine-based formulations have also been proposed for scar management [7]. Moreover, various nanoformulations have been developed as nanodelivery systems for growth factors, antibiotics and anti-inflammatory agents to overcome the limitations of their topical application [7]. Nevertheless, control of cutaneous hypertrophic scars and healing after burn injury is still challenging and new strategies are under investigation.

It is worth noting that hypertrophic scarring phenomena concern a complex scenario, comprising abnormal fibroblasts, keratinocytes and altered signaling cross-talks [8]. 

Previously, our group evaluated the antiproliferative effect of IMQ on human fibroblasts, demonstrating that IMQ inhibits the proliferation and induces the apoptosis of normal skin and HS fibroblasts [9]. The antiproliferative effect on human fibroblasts and immortalized non-tumorigenic keratinocyte cell line was enhanced when IMQ was incorporated into a new nanoformulation named β-cyclodextrin-based nanosponges (NS). NS are cross-linked polymer nanoparticles, having cyclodextrin units as building blocks. They are obtained by the reaction of β-cyclodextrins and cross-linking agents to form a polymeric network [10]. NS are solid biocompatible nanoparticles proposed as drug delivery systems to achieve drug solubilization, protection from degradation, prolonged and controlled release, enhanced permeability and bioavailability [10,11,12]. 

The ability of the cyclodextrin-based NS to enhance the drug activity was already demonstrated with a number of molecules with various structures and pharmacological activity [13,14,15]. 

The present work is aimed at developing a topical semisolid formulation, containing IMQ-loaded NS as a drug reservoir to obtain a sustained and controlled release of IMQ to the skin.

For this purpose, IMQ-loaded NS will be prepared and dispersed in a hydrogel suitable for dermal application. The formulation will be characterized in vitro and compared with IMQ inclusion complexes prepared with (2-hydroxy)propyl β-cyclodextrin (HPβCD) and carboxymethyl β-cyclodextrin (CMβCD). We selected these two β-CDs because, in preliminary experiments, they showed the best IMQ complexation capability in comparison with other β-CD derivatives.

Finally, biological assays will be carried out on cell lines to evaluate the anti-hypertrophic scarring potential of the βCD NS hydrogel.

## 2. Materials and Methods

Unless otherwise stated, the materials employed were purchased from Sigma-Aldrich (St. Louis, MO, USA).

HPβCD (degree of substitution (DS) ≈ 4.5) was a gift of Roquette (Lestrem, France) and the CMβCD (DS ≈ 4.3–4.5) were synthesized as previously reported in [16]. IMQ was purchased from In VivoGen (San Diego, CA, USA).

### 2.1. Synthesis of β-Cyclodextrin-Based Nanosponges

The β-cyclodextrin-based nanosponges (NS) were synthetized by cross-linking β-cyclodextrin with pyromellitic dianhydride as a cross-linking agent in a 1:4 molar ratio. Briefly, 6.108 g of β-cyclodextrin was dissolved in 25 mL of anhydrous dimethyl sulfoxide. Then, 6.3 mL of triethylamine and 4.695 g of pyromellitic dianhydride were added under vigorous stirring, obtaining a solid phase. Subsequently, the solid NS was ground in a mortar and washed with an excess of deionized water through Buchner filtration. An additional purification step, consisting of Soxhlet extraction in acetone, was performed for 24 h. Finally, the NS were dried at room temperature to obtain a coarse powder.

### 2.2. Preparation of Imiquimod Inclusion Complexes

To prepare the cyclodextrin inclusion complexes (IMQ–HPβCD and IMQ–CMβCD), a weighted amount of IMQ was finely suspended in a water solution containing an equimolar amount of HPβCD or CMβCD, respectively. The aqueous suspensions were then stirred at room temperature in the dark for 24 h. After centrifugation (5000 rpm, 10 min), the supernatant was freeze-dried using a Modulyo freeze-drier (Edwards, Burgess Hill, UK). To determine the IMQ percentage in the cyclodextrin complexes, a weighted amount of freeze-dried inclusion complex was dissolved in methanol. After sonication for 15 min, the sample was diluted with methanol and centrifugated, and the supernatants were analysed by High-Performance Liquid Chromatography (HPLC) for a quantitative determination of imiquimod (see Section 2.3).

### 2.3. HPLC Quantitative Determination of Imiquimod

For the quantitative determination of IMQ, an HPLC method was tuned. The analyses were carried out using a Perkin Elmer system (Perkin-Elmer, Shelton, CT, USA) under isocratic conditions. Analyses were performed using an Agilent TC C_18_ column (250 mm × 4.6 mm × 5 µm; Agilent Technologies, Santa Clara, CA, USA) tempered at room temperature. The mobile phase consisted of acetonitrile:acetate buffer (pH 4.0, 0.05 M):triethylamine (30:69.85:0.15 *v*/*v*), the flow rate was 1 mL/min and the detection wavelength was set a 242 nm. Before use, the mobile phase was filtered through a 0.45-μm-pore-size membrane filter and degassed.

The external standard method was used for the calculation of the drug content. For this purpose, about 1 mg of IMQ was weighted and dissolved in methanol in a volumetric flask to get a stock solution. This solution was diluted in the mobile phase, providing a series of calibration solutions, subsequently injected into the HPLC system (Perkin-Elmer, Shelton, CT, USA). The calibration curve was created by plotting the IMQ standard peak area vs. the corresponding drug concentration. A linear calibration curve was obtained in the 0.5–25 μg/mL concentration range with a regression coefficient of 0.999.

### 2.4. Characterization of Imiquimod Inclusion Complexes

The inclusion of IMQ in the β-cyclodextrin derivatives (either HPβCD or CMβCD) was confirmed by phase solubility studies, differential scanning calorimetry (DSC) and attenuated total reflection–Fourier transformed infrared (ATR–FTIR) analyses.

#### 2.4.1. Phase Solubility Studies

Phase solubility studies were carried out according to the Higuchi–Connors method [17]. An excess of IMQ (15 mg) was added to a series of aqueous solutions (5 mL) containing increasing concentrations of HPβCD or CMβCD, respectively, from 0 to 10 mM. The samples were stirred in the dark at room temperature for 24 h. The pH of all the solutions used in the solubility test was measured with a glass electrode of an Orion pHmeter (Orion, Jacksonville, FL, USA) and was between 6.9–7.1 at 25 ± 1 °C. After equilibration, the aqueous suspensions were centrifuged and the IMQ content in the supernatant was determined by HPLC.

The phase solubility diagram was constructed by plotting the total molar concentration of IMQ against the molar concentration of cyclodextrin (CD). 

Stability constants (*K_st_*) from the phase solubility diagram were calculated using the Equation (1):

(1) $$K_{st}=\frac{slope}{S_{0}\left(1-slope\right)}$$

where *S*_0_ represents the solubility of IMQ in the absence of CD. The slope was determined from the initial linear part of the concentration curves of IMQ.

The complexation efficiency (CE) is the concentration ratio between cyclodextrin in a complex and free cyclodextrin, and it was calculated from the phase-solubility diagrams, using Equation (2):

(2) $$\mathrm{CE}=K_{st}S_{0}$$

#### 2.4.2. Differential Scanning Calorimetry Analysis 

Thermal analyses were carried out with differential scanning calorimetry (DSC). The instrument used was a Perkin Elmer DSC/7 differential scanning calorimeter (Perkin-Elmer, Shelton, CT, USA) equipped with a TAC 7/DX instrument controller (Perkin-Elmer, Shelton, CT, USA). Indium was used for melting point and fusion heat calibration. In the 30–330 °C temperature range, a 10 °C/min heating rate was employed. Standard aluminium sample pans (Perkin-Elmer, Shelton, CT, USA) were used, and an empty pan was used as a reference standard. Analyses were performed in triplicate on 3-mg freeze-dried samples or physical mixtures under nitrogen purge.

#### 2.4.3. ATR–FTIR Spectroscopy Analysis

ATR–FTIR spectra of samples and physical mixtures were recorded on a Perkin Elmer Spectrum 100 FT-IR (Perkin-Elmer, Shelton, CT, USA) in the region of 4000–650 cm^−1^. Data acquisition was done using spectrum software version 10.03.05 Perkin Elmer Corporation (Perkin-Elmer, Shelton, CT, USA).

### 2.5. Preparation of Imiquimod-Loaded Nanosponges

For the preparation of IMQ-loaded NS, IMQ was added to the preformed NS, synthetized as described in Section 2.1. Firstly, a top-down method was used to obtain a NS nanosuspension. A weighted amount of the NS coarse powder was suspended in a saline solution (NaCl 0.9% *w*/*v*) at the concentration of 10 mg/mL. The suspension was then dispersed using a high shear homogenizer (Ultraturrax^®^, IKA, Konigswinter, Germany) for 10 min at 24,000 rpm. Then, the sample underwent high pressure homogenization (HPH) for 90 min at a back-pressure of 500 bar, using an EmulsiFlex C5 instrument (Avestin, Mannheim, Germany), to further reduce the size of the NS at nanometric range. The blank NS aqueous nanosuspension was purified by dialysis (Spectra/Por, cellulose membrane, cut-off 12,000 Da; Spectrum Laboratories, Rancho Dominguez, CA, USA) to eliminate potential synthesis residues and stored at 4 °C. 

IMQ-loaded NS were obtained by adding a known amount of IMQ to the aqueous nanosuspension of the preformed NS (10 mg/mL) at a 1:5 weight ratio. The mixture was stirred at room temperature in the dark for 24 h and subsequently centrifuged (4000 rpm) to separate the unloaded IMQ.

Blank and IMQ-loaded NS were freeze-dried using a Modulyo freeze-drier (Edwards, Crawley UK) to obtain a powder.

### 2.6. Preparation of IMQ Physical Mixtures

Binary physical mixtures of IMQ with the two β-cyclodextrin derivatives, HPβCD or CMβCD, and the NS were prepared by mixing appropriate amounts of solid components (1:1 CD:IMQ molar ratio and 5:1 NS:IMQ weight ratio) in a glass mortar.

### 2.7. Physico-Chemical Characterization of Imiquimod-Loaded Nanosponges

The physico-chemical properties of IMQ-loaded NS were characterized in vitro. The average diameter and polydispersity index of the NS were measured by photon correlation spectroscopy, using a 90 Plus instrument (Brookhaven, NY, USA). The analyses were carried out at a 90° scattering angle at 25 °C, using an NS nanosuspension diluted with filtered distilled water. The zeta potential was determined by electrophoretic mobility using the same instruments. For the zeta potential determination, samples of diluted NS formulations were placed in the electrophoretic cell, in an approximately 15 V/cm electric field.

Transmission electron microscopy (TEM) was used to evaluate the morphology of NS with a Philips CM10 (Eindhoven, The Netherlands) instrument. NS aqueous suspensions were sprayed on Formwar-coated copper grid and air-dried before observation.

A weighted amount of freeze-dried IMQ-loaded NS was dispersed in 5 mL of acetonitrile. The sample was sonicated and, after centrifugation (15,000 rpm, 10 min), the supernatant was analyzed by HPLC to quantify the amount of IMQ loaded in the NS. 

The loading capacity of the IMQ-loaded NS was calculated using Equation (3):

Loading capacity (%) = [amount of IMQ loaded/weight of NS] × 100(3)

The encapsulation efficiency was calculated using Equation (4):

Encapsulation efficiency (%) = [amount of IMQ loaded/total amount of IMQ] × 100(4)

### 2.8. In Vitro Release Studies

In vitro drug release experiments were carried out in a multi-compartment rotating cell, comprising a donor chamber separated by a cellulose membrane (cut-off = 12,000 Da) from a receiving compartment. One milliliter of the different IMQ formulations (CD complexes, IMQ-loaded NS), at the drug concentration of 1 mg/mL, was placed in the donor chamber. The receiving phase, containing saline phosphate buffer 0.05 M (pH 5.5), was withdrawn at regular intervals and replaced with the same amount of fresh buffer. Quantitative determination of IMQ in the receiving phase samples was carried out by the HPLC method, as described in the previous paragraph. Data were expressed as % of IMQ released over time. An IMQ suspension at the concentration of 1 mg/mL was used as control.

The in vitro release profiles were fitted to four distinct kinetics models to determine which one exhibited the highest correlation with the experimental release results. The zero-order kinetic model was obtained by plotting cumulative % drug release vs. time, the first-order kinetic model by plotting log of % drug remaining vs. time, the simplified Higuchi model by plotting cumulative % drug release vs. square root of time, and the Korsmeyer–Peppas model by plotting log cumulative % drug release vs. log time. For each model, the rate constant and correlation values were obtained by applying a linear regression fit.

### 2.9. Preparation and Characterization of Hydrogel Formulations

To prepare a hydrogel, hydroxyethyl cellulose was dispersed in saline solution (NaCl 0.9% *w*/*v*) at the concentration of 1.5% *w*/*v* under magnetic stirring. IMQ-loaded hydrogels were obtained by dispersing the freeze-dried IMQ–CD complexes or IMQ-loaded NS in the preformed hydroxyethyl cellulose gels. The pH values of the hydrogels were recorded at room temperature using a pHmeter Orion model 420A. The viscosity of IMQ-loaded hydrogels was determined at 25 °C using a Ubbelohde capillary viscosimeter (Schott Gerate, Mainz, Germany). 

### 2.10. In Vitro Permeation Study 

In vitro IMQ permeation studies were performed using a vertical diffusion Franz cell. The Franz cell consists of a donor compartment, in which the formulation was placed, and a receiving compartment containing the receiving phase, separated by a membrane. Purposely prepared slices of pig ear skin were used as membranes for the in vitro permeation tests. The pig skin was purchased from a local slaughterhouse. Skin slices with a thickness of 1 mm were isolated with a dermatome from the inner side of pig ears and were then frozen at −18 °C. Before the experiments, the skin was equilibrated in saline solution (NaCl 0.9% *w*/*v*) added with sodium azide (0.01% *w*/*v*) at 25 °C for 30 min to preserve its integrity. After washing with saline solution, the skin layer was inserted between the two compartments of the vertical Franz cell, with the stratum corneum (SC) side facing towards the donor chamber. The receptor phase was composed of a phosphate saline buffer 0.05 M (pH 5.5) containing sodium dodecyl sulphate (SDS) 0.1% as a solubilizer. Into the donor compartment was placed 1 mL of hydroxyethyl cellulose (1.5% *w*/*v*) gel containing various IMQ formulations (IMQ–CMβCD, IMQ–HPβCD and IMQ-loaded NS) at the drug concentration of 1 mg/mL. The donors were applied for 48 h at infinite dose. The receiving phase was withdrawn at regular times and replaced with the same amount of fresh receiving medium. Drug permeation was evaluated, determining the cumulative amount of IMQ reaching the receiving phase over time by HPLC analysis.

### 2.11. Determination of Imiquimod in Skin Samples

The amount of IMQ that accumulated in the skin was evaluated at the end of the in vitro permeation studies. Ear pig skin used for the in vitro permeation tests underwent solvent extraction to evaluate the accumulation of IMQ in the SC, epidermis and dermis, respectively. At the end of the permeation experiments, the application site of the pig skin was washed with saline solution to remove the formulation on the surface. Then, the skin was dried with cotton wool and the SC was removed by tape-stripping the skin with 25 adhesive cellophane tapes. The stripping tapes were transferred into glass vials containing 5 mL of methanol to extract IMQ. The remaining stripped skin (epidermis without SC plus dermis) was then heated (hairdryer for 60 s) and separated into the epidermis and dermis with the help of a spatula. The two parts were cut with a scalpel into small pieces that were placed in 5 mL of methanol for solvent extraction. Extractions were carried out in an ultrasonic bath at 40 °C for 15 min, and repeated three times. 

### 2.12. Biological Studies

#### 2.12.1. Cell Cultures

Fibroblasts were isolated from cutaneous normal human skin biopsies (*N* = 3) taken from patients after having received individual informed consent (approved by the Ethical Committee of the AOU “Città della Salute e della Scienza di Torino”—CTO Hospital, protocol n. 0041451) during reconstructive plastic procedures at the Turin Burn Centre.

Briefly, the tissue was minced and incubated overnight in Dulbecco’s modified Eagle’s medium (DMEM) (Gibco, Invitrogen, Waltham, MA, USA) supplemented with 100 U/mL penicillin and 100 mg/mL streptomycin. Afterwards, the biopsies were incubated in 2 mg/mL Dispase II (Roche, Manheim, Germany) at 37 °C and 5% CO_2_ for 2 h. The dermis, obtained after epithelial layer removal, was incubated for 1 week in 24-well plates at 37 °C and 5% CO_2_ in DMEM supplemented with 10% Fetal Bovine Serum (FBS) (Sigma, St. Louis, MO, USA) and antibiotics.

Once an adequate growth of fibroblasts had been detected, the cells were trypsinized for subcultures in 75-cm^2^ tissue culture flasks. For stimulation experiments, fibroblasts were used at 4–6 passages of culture.

#### 2.12.2. MTT Colorimetric Assay

The viability of the fibroblasts was measured by the 3-(4,5-dimethylthiazol-2-yl)-2,5-diphenyltetrazoliumbromide (MTT) assay (Roche, Risch, Switzerland). Fibroblasts were seeded at a density of 1 × 10^4^ cells/well in a 24-well plate. After 2 h, the cells were either incubated with the indicated compounds or left untreated. Cell proliferation analysis was performed in triplicate after 24, 48, 72 and 96 h of treatment by spectrophotometric readings at 570 nm using an Eppendorf BioPhotometer (Eppendorf, Hamburg, Germany).

Data are shown as the optical density or as a percentage of the average proliferation rate compared to the control/unstimulated cells or blank NS.

#### 2.12.3. In Vitro Scratch Wound Assay

The assay was performed as previously described by Bastiancich et al. [9]. Briefly, the fibroblasts were seeded in 4-well chamber slide (Thermo SC NUNC, Roskilde, Denmark) at a concentration of 1 × 10^4^ cells. Confluent monolayers were synchronized for 2 h in DMEM containing 0.5% FBS, and wounded by removing a 500–800-µm wide strip of cells across the well with a standard 200 µL pipette tip. The wounded culture wells were washed with phosphate-buffered saline (PBS) to remove any non-adherent cells, and treated with free IMQ (5 µg/mL) or IMQ–CDNS (5, 25 µg/mL) or blank NS. The wound area was observed and photographed by an optical microscope (Axiovert, ZEISS, Jena, Germany) at 0, 24, and 72 h.

### 2.13. Statistical Analysis

Data are expressed as the mean ± standard error of the mean (SEM). Statistical significance between the experimental groups was determined by two-way ANOVA followed by Bonferroni post tests (GraphPad Prism version 4.00 for Windows, GraphPadSoftware, San Diego, CA, USA).

## 3. Results and Discussion

The topical delivery of IMQ is highly challenging because of its very low solubility in aqueous media (0.01 mM) and skin permeation capability. Indeed, despite the low molecular weight (MW 240.30 g/mol) and logP value of 2.6, IMQ shows very poor skin penetration [18].

The aim of this work was the development of a nanomedicine-based topical formulation for the sustained delivery of IMQ through the skin, for the potential control of the scarring process and the treatment of aberrant wounds (e.g., HS).

Interestingly, CD complexation can enhance drug solubility and skin permeation when it is hampered by poor drug solubility. Here, the effect of two β-CD derivatives, HPβCD and CMβCD, on the solubility of IMQ was studied by phase solubility tests. 

### 3.1. Characterization of Imiquimod Inclusion Complexes

**Figure 1:**
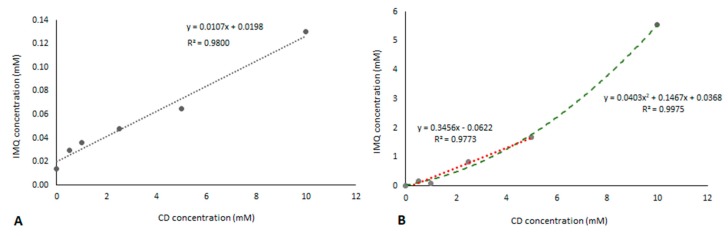
Phase solubility tests of the imiquimod (IMQ) inclusion complexes of (2-hydroxy)propyl β-cyclodextrin (HPβCD) (**A**) and carboxymethyl β-cyclodextrin (CMβCD) (**B**).

The results of the phase solubility study (Figure 1A,B) show that the solubility of IMQ increases as a function of HPβCD or CMβCD concentrations. The linear relationships of IMQ solubilities with the CD concentrations were found. The phase solubility profile obtained was an “A-type” diagram, according to the Higuchi and Connors classification [17]. 

The slopes of the curves of both complexes were lower than one, demonstrating the formation of 1:1 inclusion complexes. In the case of CMβCD, the solubility isotherm shows an A_p_ type curve, suggesting the formation of other than 1:1 complexes.

The intrinsic solubility of IMQ (*S*_0_ value) was 0.01 mM.

The calculated apparent complex stability constant (*K_st_*) of the IMQ–HPβCD inclusion complex was 779.8 M^−1^ and the complexation efficiency (CE) was 0.01 (Table 1).

A higher complexation capability was observed for CMβCD, showing an apparent stability constant (*K_st_*) of 10,552.2 M^−1^ and a complexation efficiency of 1.25 (Table 1). Consequently, CMβCD showed the best IMQ solubilization efficiency. The higher complexation capability of CMβCD than that of HPβCD was already demonstrated with hydrocortisone by Loftsson [19]. 

Interestingly, to explain the phase solubility results we might also hypothesize the presence of a second complexation contribution due to the electrostatic interaction between the IMQ positive amino group and negative carboxymethyl groups of CD. This second interaction might increase the complex stability in addition to the drug inclusion in the CD cavity.

The cavity extension, provided by an additional external interaction, can play a role in the complex stabilization, as previously shown with alkyl-substituted cyclodextrins [20].

**Figure 2:**
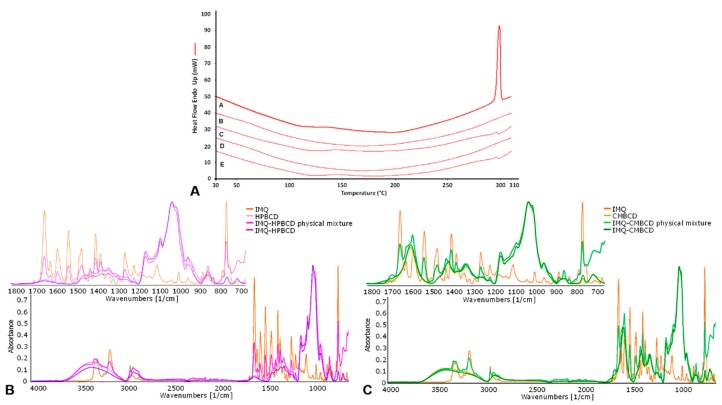
(**A**) Differential scanning calorimetry (DSC) thermograms of imiquimod (IMQ) and IMQ inclusion complexes: (A) IMQ, (B) IMQ–HPβCD, (C) IMQ–HPβCD physical mixture, (D) IMQ–CMβCD, (E) IMQ–CMβCD physical mixture; (**B**) Attenuated total reflection–Fourier transformed infrared (ATR–FTIR) spectra of IMQ and HPβCD complex; (**C**) Attenuated total reflection–Fourier transformed infrared (ATR–FTIR) spectra of IMQ and CMβCD complex.

The two inclusion complexes were freeze-dried to obtain a powder and investigated by DSC and ATR–FTIR analyses. DSC thermograms of IMQ and IMQ inclusion complexes were reported in Figure 2A.

The lack of an endothermic peak related to the drug melting at approximately 295 °C in the inclusion complex thermograms showed the interaction between IMQ and HPβCD or CMβCD. After complexation, the drug is molecularly dispersed in the cyclodextrin cavity without the possibility to form crystals. The absence of the melting peak of the drug complexed with HPβCD was already described by Ramineni et al. [21]. Conversely, a detectable peak at approximately the IMQ melting temperature was observed in the physical mixture thermograms.

The ATR–FTIR data of the drug after the formulation with the two cyclodextrins confirmed the molecular interaction. Indeed, the disappearance and the shifts of the guest molecule spectral featured in the two inclusion complexes (IMQ–HPβCD or IMQ–CMβCD) underline the inclusion of imiquimod in the CD cavity (Figure 2B,C).

CDs have been previously applied to optimize the dermal delivery of drugs for local use [22] and CD-modified hydrogels have been developed for the sustained release of drugs for topical therapy [23]. In addition, it was shown that cyclodextrins can act as penetration enhancers modifying the skin barriers. 

Cyclodextrin polymers can also be used to favor penetration into the skin. Indeed, the loading of a drug into a nanodelivery system is a strategy to enhance drug permeation across the skin [24,25,26,27]. 

### 3.2. Physico-Chemical Characterization of Imiquimod-Loaded Nanosponges

We selected this NS architecture for IMQ loading because they are swellable in aqueous media and contain free carboxylic groups in the polymer matrix that can electrostatically interact with the drug. To formulate IMQ in NS, firstly it was necessary to prepare an aqueous NS nanosuspension. Using a purposely tuned HPH method has enabled obtaining NS in the nanoscale range size with almost homogenous size distribution.

Table 2 reports the physico-chemical characteristics of blank NS in comparison with the IMQ-loaded formulation.

The blank NS showed average diameters of about 400 nm and negative zeta potential (about −30 mV). The negative zeta potential of the blank and IMQ-loaded NS was high enough to guarantee the physical stability of the colloidal nanoformulation, preventing aggregation phenomena over time. The incorporation of IMQ in the NS did not significantly change the physico-chemical properties of NS, showing that the drug is not absorbed on the NS surfaces, but is almost encapsulated inside the polymer matrix. Indeed, NS were able to load IMQ to a high extent, with 14.2% drug loading and 96.5% encapsulation efficiency.

**Figure 3:**
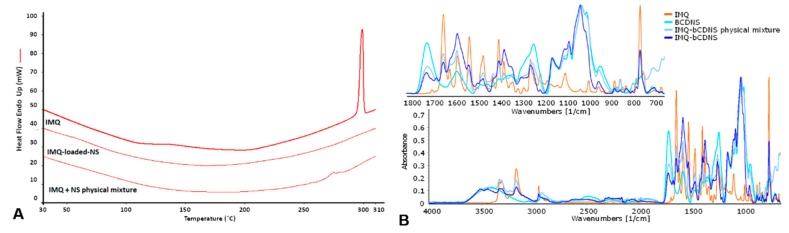
(**A**) DSC thermograms of IMQ (**A**), IMQ–NS physical mixture (**B**) and IMQ-loaded NS (**C**); (**B**)ATR–FTIR spectra of IMQ and IMQ-loaded NS.

Subsequently, the incorporation of IMQ in the NS nanostructure was studied by ATR–FTIR and DSC analyses. The ATR–FTIR spectra of IMQ as such and loaded in NS are reported in Figure 3, in comparison with blank NS.

The lack of an endothermic melting peak of IMQ (at approximately 295 °C) in the DSC thermogram of IMQ-loaded NS indicated that IMQ is molecularly incorporated into the NS matrix and unable to crystallize (Figure 3A). On the other hand, the physical mixture of IMQ with NS shows two main endothermic peaks in the temperature range of 284–288 °C. The shift of the melting peaks of the drug to a lower temperature may indicate the physical interaction between NS and IMQ. Indeed, a non homogeneus system formed but two different phases are present, indicating that the sample is not 100% drug. This thermal behavior is in agreement with previous results obtained using other drugs loaded in NS formulations [28,29,30].

The physical interaction between IMQ and NS was also confirmed by ATR–FTIR analyses, as shown in Figure 3B. The spectra showed the disappearance and the shift of the drug peaks after loading in the NS polymer matrix.

**Figure 4:**
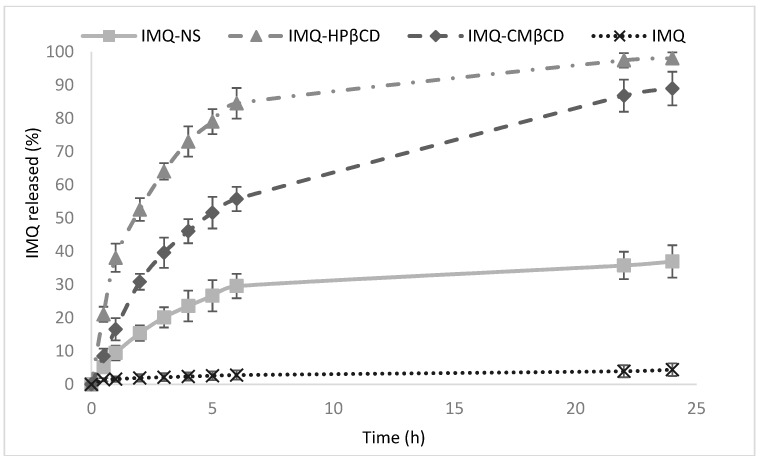
In vitro release profile of IMQ from the NS in comparison with the cyclodextrin (CD) inclusion complexes and free IMQ.

Figure 4 shows the in vitro release kinetics of IMQ from the NS at pH 5.5, a value selected to simulate the skin pH in a perspective permeation study with Franz cells. A slow and prolonged in vitro release profile of the drug was observed. Less than 35% of IMQ was released after 24 h. Interestingly, no initial burst effect was observed, confirming the incorporation of IMQ in the NS matrix and not its adsorption on the nanoparticle surface. The release kinetics of IMQ from NS exhibited correlation with the Korsmeyer–Peppas kinetics model, suggesting that the release from NS is influenced by the drug diffusion from a swellable polymer matrix.

For comparison, the diffusion of IMQ from the inclusion complexes was investigated in vitro.

The complexation of IMQ with the two CDs increased the apparent drug solubility and favored the diffusion of IMQ in the receiving compartment. Indeed, 85% of IMQ was released after 6 h from the IMQ–HPβCD complex, and 56% was released from the IMQ–CMβCD complex in the experimental conditions (pH 5.5). On the other hand, the percentage of IMQ diffused from the drug suspension was only 4% after 24 h.

### 3.3. In Vitro Permeation Study

IMQ inclusion complexes and IMQ-loaded NS were formulated into a vehicle suitable for skin administration. For this purpose, IMQ–HPβCD, IMQ–CMβCD and IMQ-loaded NS were incorporated in hydroxyethyl cellulose (1.5% *w*/*v*) hydrogel. 

Hydroxyethyl cellulose is a non-ionic, water-soluble polymer widely used in topical pharmaceutical formulations and cosmetic products on the market. It is nontoxic and nonirritant and admitted by the regulatory agencies. The pH and viscosity (pH 6.5 and 35 cP) of the hydrogels were tuned taking into account skin application for wound treatment.

**Figure 5:**
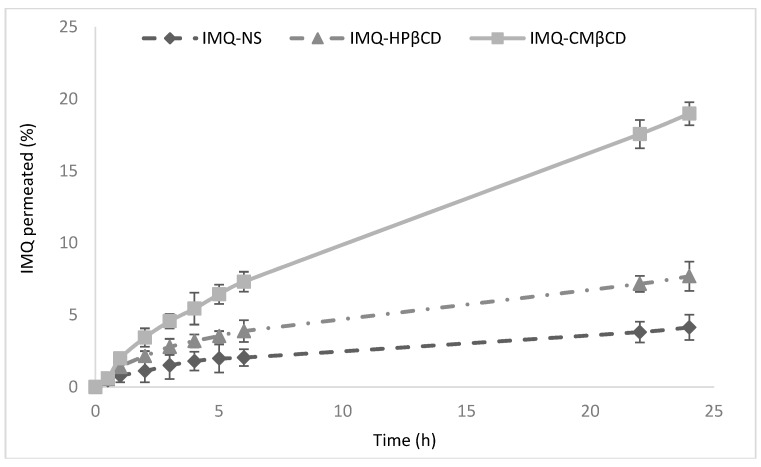
In vitro permeation profiles of IMQ-loaded NS and IMQ complexes through pig skin.

Then, in vitro permeation studies were carried out using the three IMQ formulation-loaded hydrogels. The Franz cell permeation assays consisted of the application of the IMQ hydrogel formulations on pig ear skin at infinite dose [31]. The in vitro permeation profiles of IMQ-loaded NS and IMQ complexes through pig skin are reported in Figure 5.

The complexation with HPβCD and CMβCD enhanced the amount of IMQ permeated across the skin. 

These results are probably related to the increase of drug availability at the surface of the skin. 

Sigurdadottir and Loftsson [19] demonstrated that cyclodextrins can act as a drug carrier, delivering lipophilic drugs to the skin surface where they can penetrate into the skin with an increased flux. Various studies have shown that CDs in topical formulations enhance drug penetration into and drug flux through the skin from aqueous vehicles [32]. Indeed, CDs are able to solubilize poorly soluble drugs in the aqueous external medium, increasing the drug concentration gradient over the lipophilic biological membrane [33,34].

Here, as expected, the results obtained showed that the flux of IMQ increased when it was in a complexed form. In particular, about 7.7% of IMQ passed through the skin layer after 24 h from IMQ–HPβCD, whereas, when IMQ was complexed with CMβCD, the percentage of IMQ permeation reached 19% after 24 h. The enhancement of IMQ permeation seems to depend on the type of CD used in addition to the available amount of the drug in the solution on the surface of the skin. The higher flux of IMQ complexed with CMβCD may be partially explained by the action of CMβCD on the skin itself [19]. Conversely, the permeation of IMQ through the skin from the IMQ-loaded NS was slower than those of the two complexes. Indeed, 4% of IMQ permeated the skin after 24 h. These results are in agreement with the in vitro release kinetics of IMQ from NS. This sustained and prolonged release can represent an advantage for IMQ administration in wounds, allowing the decrease in the number of applications and reduction of off-target side effects due to application on large superficial areas.

Subsequently, the amount of IMQ accumulated in the skin was determined at the end of the in vitro permeation studies.

Table 3 shows the permeation parameters for IMQ inclusion complexes and IMQ-loaded NS.

Higher skin retention after 24 h was found for IMQ-loaded NS, which showed a total accumulated amount of approximately 105 μg/cm^2^ of IMQ. It is worth noting that nanomedicine-based systems can favor interactions at the sub-atomic level, with the skin facilitating the delivery through the biological membrane [35].

Recently, a number of nanomedicine-based formulations have been studied for the treatment of wound healing and burns [7]. Ma et al. demonstrated that IMQ incorporated in transethosomes increased drug permeation and its local accumulation efficiency either in vitro or in vivo [36].

Based on these premises, we selected an IMQ-loaded NS hydrogel as a more suitable formulation for the topical treatment of HS. Therefore, further investigations on the skin distribution and biological assays were carried out only on the IMQ-loaded NS hydrogel.

The distribution of IMQ loaded in NS in the different layers of the skin, i.e., SC, epidermis and dermis was studied following application on the skin of the hydrogel containing IMQ-loaded NS for 24 and 48 h.

**Figure 6:**
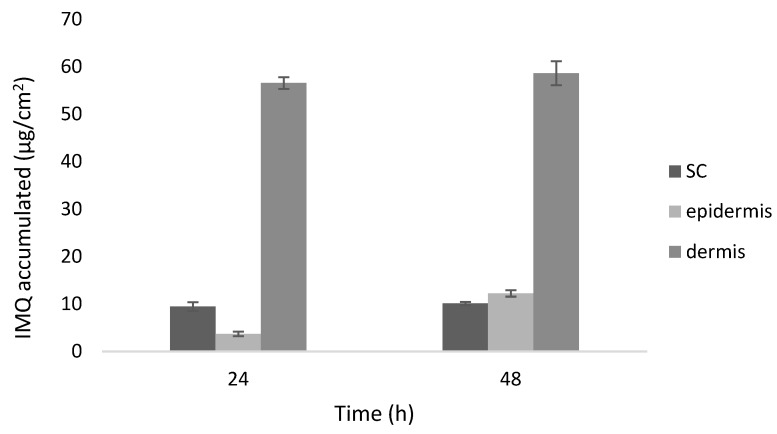
In vitro accumulation of IMQ in the stratum corneum (SC), epidermis, and dermis following application on the pig skin of a hydrogel containing IMQ-loaded NS for 24 and 48 h.

The results showed that IMQ accumulated mainly in the dermis (60 µg/cm^2^ of IMQ after 48 h), while, in the SC and epidermis, the concentration of IMQ accumulated was 10 and 12 µg/cm^2^, respectively (Figure 6).

This accumulation profile might play a key role in controlling fibroblast activity in wounds.

### 3.4. Biological Studies

**Figure 7:**
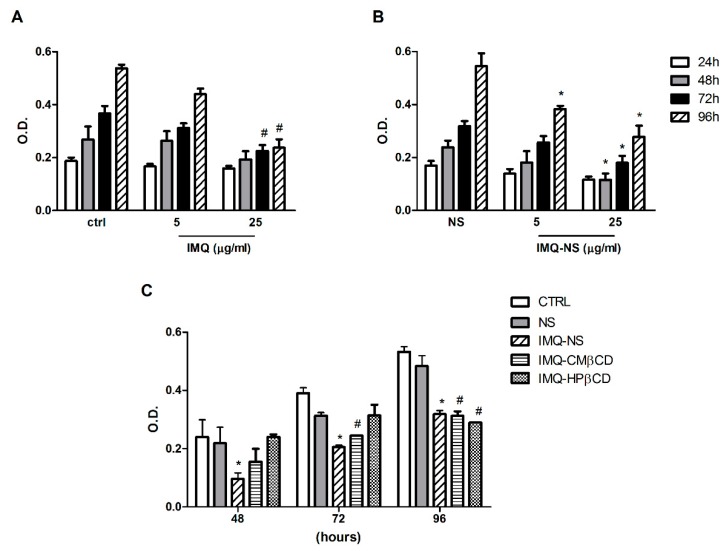
Effect of free IMQ, IMQ–NS and IMQ–CD complexes on the proliferation of normal skin-derived fibroblasts. Fibroblasts were left untreated or treated with 5 and 25 µg/mL of free IMQ, blank NS (NS) and IMQ–NS (**A**,**B**) or with NS, IMQ–NS, IMQ–CMβCD and IMQ–HPβCD at 25 µg/mL (**C**) for the indicated times. Data are shown as the optical density (O.D.). Cell proliferation is presented as mean ± SEM of three independent experiments. # *p* < 0.05 vs. untreated cells, * *p* < 0.05 vs. NS as appropriate, by two-way ANOVA followed by Bonferroni post tests.

The effects of IMQ formulations on fibroblast proliferation were evaluated using the MTT assay. First, the cells were exposed to IMQ–NS and free IMQ, at two different concentrations (5 and 25 µg/mL), for up to 96 h (Figure 7A,B). Untreated cells and blank NS (NS) were used as controls. Our results demonstrate that the treatment with blank NS did not significantly affect cell viability, confirming the safety of this nanocarrier. IMQ–NS presented antiproliferative activity at low doses (5 µg/mL), significantly evident at 96 h (Figure 7B). At a higher concentration (25 µg/mL), both IMQ–NS and free IMQ showed a good antiproliferative effect; however, IMQ–NS was effective as early as at 48 h (Figure 7A,B). As can be seen in Figure 7C, the IMQ–CD complexes (IMQ–CMβCD and IMQ–HPβCD) also exhibited an antiproliferative effect on fibroblasts, although IMQ–NS showed a superior effect. 

**Figure 8:**
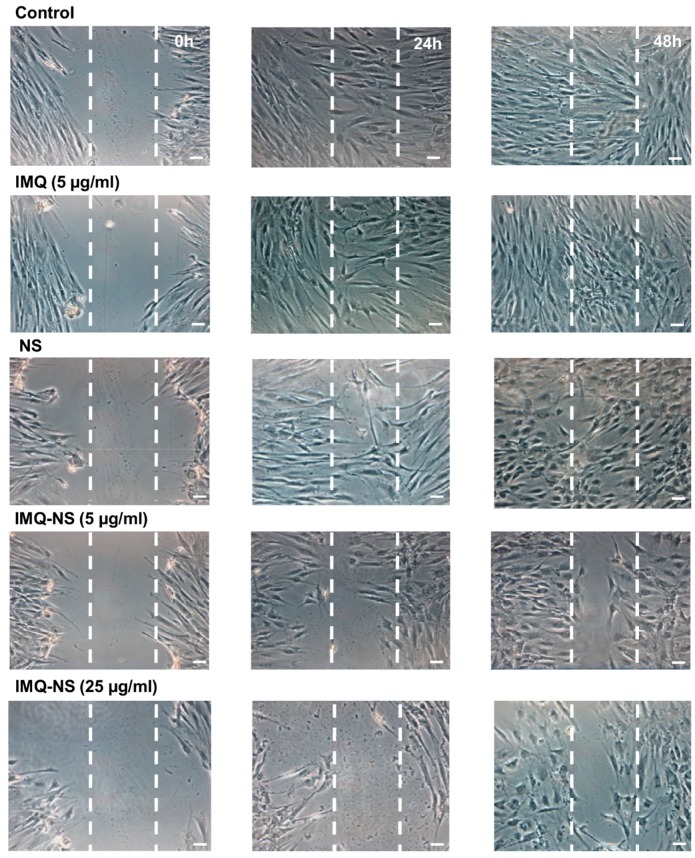
Inhibitory effect of IMQ–NS in a scratch wound assay. The figure shows micrographs of the extent of closure obtained under control conditions compared to those with free IMQ (5 µg/mL), NS or IMQ–NS (5 and 25 µg/mL) after 24- and 48-h treatment. The fibroblasts were synchronized for 2 h, wounded and treated as indicated, and phase-contrast microscopy pictures were taken of the wounded area (scale bar 100 µm). One representative experiment out of three is shown.

Next, we choose to investigate the effect of IMQ–NS on the migration of fibroblasts in cell culture using the scratch wound assay. The scratch area of cells treated with 25 µg/mL of IMQ–NS appeared to have a lower number of fibroblasts within the scratch region compared to empty NS- treated cells. Moreover, while with free IMQ at 5 µg/mL, a complete closure of the scratch area was observed at 48 h, IMQ–NS prevented healing at the same dose (Figure 8). Taken together, the results suggest that encapsulation in nanosponges increases the antiproliferative and antimigratory activity of IMQ.

## 4. Conclusions

To conclude, IMQ can be complexed with HPβCD and CMβCD, forming stable inclusion complexes able to enhance its aqueous solubility and skin permeation capability. 

NS can incorporate and store the drug to a good extent. IMQ loaded in NS can be released with prolonged and controlled release kinetics. Loaded NS could act as a drug reservoir dispersed in the hydrogel, able to slowly deliver IMQ through the skin, favoring dermal accumulation. This behavior could allow for a reduction in the number of applications, enhancing patient compliance. Moreover, a decrease in the irritating effect on skin might be obtained.

These results might pave the way to develop an innovative topical nanomedicine formulation for the prevention and treatment of hypertrophic scars.

## Figures and Tables

**Table 1:** Stability constant (M^−1^) and complexation efficiency (CE) of imiquimod (IMQ) inclusion complexes. Please add the full name.

CD Complex	Stability Constant (M^−1^)	CE
**IMQ–HPβCD**	779.8	0.01
**IMQ–CMβCD**	10552.2	1.25

**Table 2:** Physico-chemical characteristics of blank and IMQ-loaded nanosponge (NS) formulations.

Physico-Chemical Characteristics	Blank NS	IMQ-Loaded NS
Average diameters ± SD (nm)	405.8 ± 12.4	412.6 ± 10.2
Polydispersity index	0.18 ± 0.02	0.19 ± 0.01
Zeta potential ± SD (mV)	−32.12 ± 2.28	−29.86 ± 1.45

**Table 3:** Permeation parameters for IMQ–CD complexes and IMQ-loaded NS. Results are expressed as mean ± S.D. (*n* = 3).

In Vitro Permeation Parameters	IMQ–HPβCD	IMQ–CM–βCD	IMQ-Loaded NS
**Flux J (μg/cm^2^·h^−1^)**	1.88 ± 0.06	4.57 ± 0.08	1.02 ± 0.10
**IMQ accumulated (μg/cm^2^)**	46.87± 1.42	20.60± 2.05	105.67± 2.36
